# Perceptions and engagement of patients with chronic conditions on the use of medical cannabis: a scoping review

**DOI:** 10.1186/s40001-024-01803-w

**Published:** 2024-04-01

**Authors:** Marie-Pascale Pomey, Didier Jutras-Aswad, Jesseca Paquette, Kamilla Saadi, Mélissa Taguemout, Dina-Liza Ikene, Nathalie Arbour, Amel Zertal, Nathalie Fréjeau, Danielle Morin, Jean-Sylvain Ouellette, Kanza Alami Marrouni, Pierre Duquette

**Affiliations:** 1 University of Montreal Hospital Research Centre (CRCHUM), Montréal, QC Canada; 2 School of Public Health, Department of Health Management, Evaluation of Policy, Université de Montréal, 7101 Du Parc Avenue 3rd Floor, Montréal, QC H3N 1X9 Canada; 3 Centre of Excellence on Partnership with Patients and the Public, Montréal, QC Canada; 4 Department of Psychiatry and Addiction, Université de Montréal, Montréal, QC Canada; 5 Faculty of Medicine, Université de Montréal, Montréal, QC Canada; 6 Department of Neurosciences, Faculty of Medicine, Université de Montréal, Montréal, QC Canada; 7 Department of Anthropology, Université de Montréal, Montréal, QC Canada; 8 Multiple Sclerosis Society of Canada, Toronto, ON Canada

**Keywords:** Scoping review, Medical cannabis, Chronic disease, Chronic conditions, Patients, Perspectives, Beliefs, Knowledge, Effects

## Abstract

**Context:**

Studies generally focus on one type of chronic condition and the effect of medical cannabis (MC) on symptoms; little is known about the perceptions and engagement of patients living with chronic conditions regarding the use of MC.

**Objectives:**

This scoping review aims to explore: (1) what are the dimensions addressed in studies on MC that deal with patients' perceptions of MC? and (2) how have patients been engaged in developing these studies and their methodologies? Through these objectives, we have identified areas for improving future research.

**Methods:**

We searched five databases and applied exclusion criteria to select relevant articles. A thematic analysis approach was used to identify the main themes: (1) reasons to use, to stop using or not to use MC, (2) effects of MC on patients themselves and empowerment, (3) perspective and knowledge about MC, and (4) discussion with relatives and healthcare professionals.

**Results:**

Of 53 articles, the main interest when assessing the perceptions of MC is to identify the reasons to use MC (*n* = 39), while few articles focused on the reasons leading to stop using MC (*n* = 13). The majority (85%) appraise the effects of MC as perceived by patients. Less than one third assessed patients’ sense of empowerment. Articles determining the beliefs surrounding and knowledge of MC (*n* = 41) generally addressed the concerns about or the comfort level with respect to using MC. Only six articles assessed patients’ stereotypes regarding cannabis. Concerns about stigma constituted the main topic while assessing relationships with relatives. Some articles included patients in the research, but none of them had co-created the data collection tool with patients.

**Conclusions:**

Our review outlined that few studies considered chronic diseases as a whole and that few patients are involved in the co-construction of data collection tools as well. There is an evidence gap concerning the results in terms of methodological quality when engaging patients in their design. Future research should evaluate why cannabis’ effectiveness varies between patients, and how access affects the decision to use or not to use MC, particularly regarding the relationship between patients and healthcare providers. Future research should consider age and gender while assessing perceptions and should take into consideration the legislation status of cannabis as these factors could in fact shape perception. To reduce stigma and stereotypes about MC users, better quality and accessible information on MC should be disseminated.

**Supplementary Information:**

The online version contains supplementary material available at 10.1186/s40001-024-01803-w.

## Introduction

Chronic diseases are collectively responsible for about 70% of all deaths worldwide, placing a growing burden on individuals and healthcare systems around the world [1]. Besides this impact on mortality, their load of morbidity is not to be underestimated. In fact, as most chronic conditions still have no cure, they require lifelong follow-up, self-management, adherence to treatment and timely intervention when necessary [2]. Given their duration, some people living with chronic conditions can experience emotional stress and chronic pain, which are both associated with the development of depression and anxiety. [3]

In this context, patients with oncological and non-oncological chronic conditions should play an increasingly important role in managing their illness [4, 5]. Engaging them in the self-management of their chronic conditions could help increase their quality of life by carrying out normal roles and activities, and manage the physical and emotional impact of their illness [6–8]. Support in self-management by healthcare providers has been shown to help patients deal with their symptoms more efficiently and can also help these patients to “gain the confidence, knowledge, skills, and motivation to manage the physical, social, and emotional impacts of their disease” [9]. However, some factors, such as comorbidities or psychosocial vulnerability, can further challenge providers' care and patients' self-management. These issues can undermine patient participation in care and affect treatment adherence and attendance. [10]

Therapeutic benefits of medical cannabis (MC) have been demonstrated across a broad spectrum of medical conditions and symptoms and can act as an analgesic, anticonvulsant, and antispasmodic for a wide range of chronic conditions [11]. Chronic pain and co-occurring conditions are among the most common conditions for which cannabinoid-based products, mainly containing Δ^9^-tetrahydrocannabinol (THC) and/or cannabidiol (CBD), are used for therapeutic purposes [12], knowing, however, that there are uncertainties and controversies about the role and appropriate use of cannabis-based medicines in the management of chronic pain [13]. Other than non-prescribed cannabis, some patients have access to cannabinoid-based products to alleviate symptoms specific to their chronic disease such as Sativex^®^ for multiple sclerosis, Cesamet^®^ for chemotherapy, Marinol^®^ for AIDS and chemotherapy, and Epidiolex^®^ for epilepsy [14]. Although these products are not without risks, the “majority of reported adverse events tends to be mild and self-limiting.” [15].

Even if some studies have shown MC to be effective in managing chronic pain [16] and in improving quality of life [17], some healthcare providers may be reluctant to discuss and support patients’ use of MC due to concerns about the quality of evidence and general lack of information [18] on or lack of knowledge of MC dosing and about how to create treatment plans with MC [19].

Studying the perception of people with chronic conditions of MC and their motivations to use it should help to identify its major issues to eventually produce coherent treatment plans. Moreover, involving patients in the construction of such studies should also add relevant elements to the analysis. Indeed, directly involving patients in research, in the recruitment or tools development, could help better assess these challenges and facilitators in patients’ self-management [20, 21], since they can help identify crucial elements that could have otherwise been overlooked. [22–24].

MC is a growing interest in clinical research and for chronically ill patients. While potential benefits or harms of MC are still under research, MC is considered an option for a complementary treatment by patients [25]. In this context, knowing how and to what extent the perceptions of the population affected by oncological and non-oncological diseases are covered should shed light on potential predisposing factors enabling or not the use of MC. Finally, as this subject pertains directly to the subjectivity of patients, having this population involved in the creation of the research design and outcome measures could give insights otherwise overlooked. Consequently, it is important to observe the degree to which this population is involved in the construction of those tools.

In this context, the primary objective of this scoping review is to map out and analyze the literature on the perception and engagement of patients living with oncological and non-oncological chronic conditions regarding the use of MC. This was achieved through two distinct questions: (1) What are the dimensions addressed in studies on MC that deal with patients' perception of MC? and (2) how have patients been engaged in developing these studies and their methodologies?

Through these questions, we hope to highlight gaps in the literature and suggest avenues for future research.

## Materials and methods

We followed the phases of the flow diagram developed by the Preferred Reporting Items for Systematic Reviews and Meta-Analyses: extension for scoping reviews (PRISMA–ScR) to apply a systematic approach when conducting the review and reporting the results [26]. The scoping review was also conducted in accordance with the multistage framework outlined by Arksey and O’Malley [27] and JBI synthesis evidence [28–30], as detailed in the following sections.

### Identifying relevant studies

To initiate the literature search, we have identified six major concepts related to: patients, oncological and non-oncological chronic disease, perceptions, cannabis, medical use, and effects. The latter was added to find associated factors that could have an impact on patients’ perceptions. All descriptors are presented in Table 1. The search strategy was based on five major health and social science databases: Ovid MEDLINE, Ovid EMBASE, Elsevier Scopus, Clarivate Web of Science, and EBSCO CINAHL. The initial search was conducted on November 29, 2021 to capture publication from 2002 to 2022 and another was conducted on September 27, 2022 for publications from 2021 to 2022. Our search strategy was restricted to published and peer-reviewed literature and we did not conduct any searches in the gray literature. The search strategies are available in supplementary files (Additional file 1).

**Table 1:** Descriptors

Patients	Chronic disease	Perspectives	Cannabis	Medical use	Effects
Patient*	Chronic	Perspective*	Marijuana	Treatment*	Effect*
User*	Pain	Preference*	Cannabis	Therap*	Impact
Adult*	Disease*	Attitude*	Cannabinoid*	Alternative	Stigma*
	Symptom*	Perception*	Cannabidiol	Management	‘‘Quality of life’’
	Illness	Experience*	Tetrahydrocannabinol	Palliative	Benefit*
	Condition	Belief*	THC^a^	Complementary	Outcome*
	Disorder*	View*	CBD^b^	Medic*	Harm
		Opinion*			

The asterisk appended to certain words is utilized to facilitate database searches that generate results including various declensions or forms, such as plural form

^a^Tétrahydrocannabinol

^b^Cannabidiol

### Study selection

The inclusion and exclusion criteria are detailed in Table 2. The references were managed with EndNote. After removing duplicates, four reviewers (J.P., D.L.I., M.T., and K.S.) independently screened articles based on their titles and abstracts using Rayyan. A pilot round was conducted with 50 references to verify the reviewers’ agreement on the inclusion and exclusion criteria based on the title and abstract of each before performing a full screening of the rest of the articles. Discrepancies were resolved through team discussion and consensus. The senior authors (M.P.P. and D.J.A.) screened articles of uncertain relevance for final inclusion or exclusion.

**Table 2:** Inclusion and exclusion criteria

	Inclusion criteria	Exclusion criteria
Subject	Perceptions of patients with a chronic disease regarding the use of therapeutic cannabis	Studies only describing the effects or efficacy of MC on patient symptoms Studies exposing only the pharmaceutical and pharmacodynamic characteristics of cannabis Studies describing the perception of health professionals or students or the public
Type of article	Original peer-reviewed and published studies	Systematic reviews, editorials, documents from the gray literature, articles that have not been peer-reviewed, and preprint articles
Population	Adults	Children or teenagers
Language	Studies published in English or French	Studies published in a language other than English or French

### Charting the data

Data from the included articles was charted on an extraction grid (Additional file 2) according to the following categories: authors, title, journal, year of publication, study location, type of chronic disease, aims of the study and method used to collect data.

### Collating, summarizing and reporting the results

We used a thematic analysis approach to identify, analyse and report patterns or themes in the literature. This approach allows adaptability and flexibility to refine or create new categorizations if needed [31, 32]. The first step in the data synthesis was to become familiar with the data by reading full texts to identify a wide range of subjects that were then grouped into main themes that assess the perceptions and engagement of patients with chronic diseases about MC [33]. Some themes were then merged to form broader categories to synthesize the information and facilitate the reporting of results. Finally, the themes were refined according to the research objectives. The final themes were then used to analyze the articles by two coders (J.P., K.S.).

We also assessed the level of patients’ involvement in the research process of the included articles. Involving patients in the research process as opposed to conducting research for or about them is the definition of Patient and Public Involvement (PPI) [33]. Patients’ involvement can testify to the quality, relevance and impact of the research by improving researchers’ transparency and accountability [33]. To evaluate the level of engagement in research, we used the continuum of patient involvement in research [34]. We considered three levels of involvement: (1) consultation of patients, which refers to asking for patients' input during the themes of identification or tool validation; (2) collaboration with patients, which corresponds to involving them in the selection and wording of items in the questionnaire or interview guide; and (3) partnership, which refers to co-constructing the tool with patients, from its development to its validation.

## Results

### Search results

**Fig. 1:**
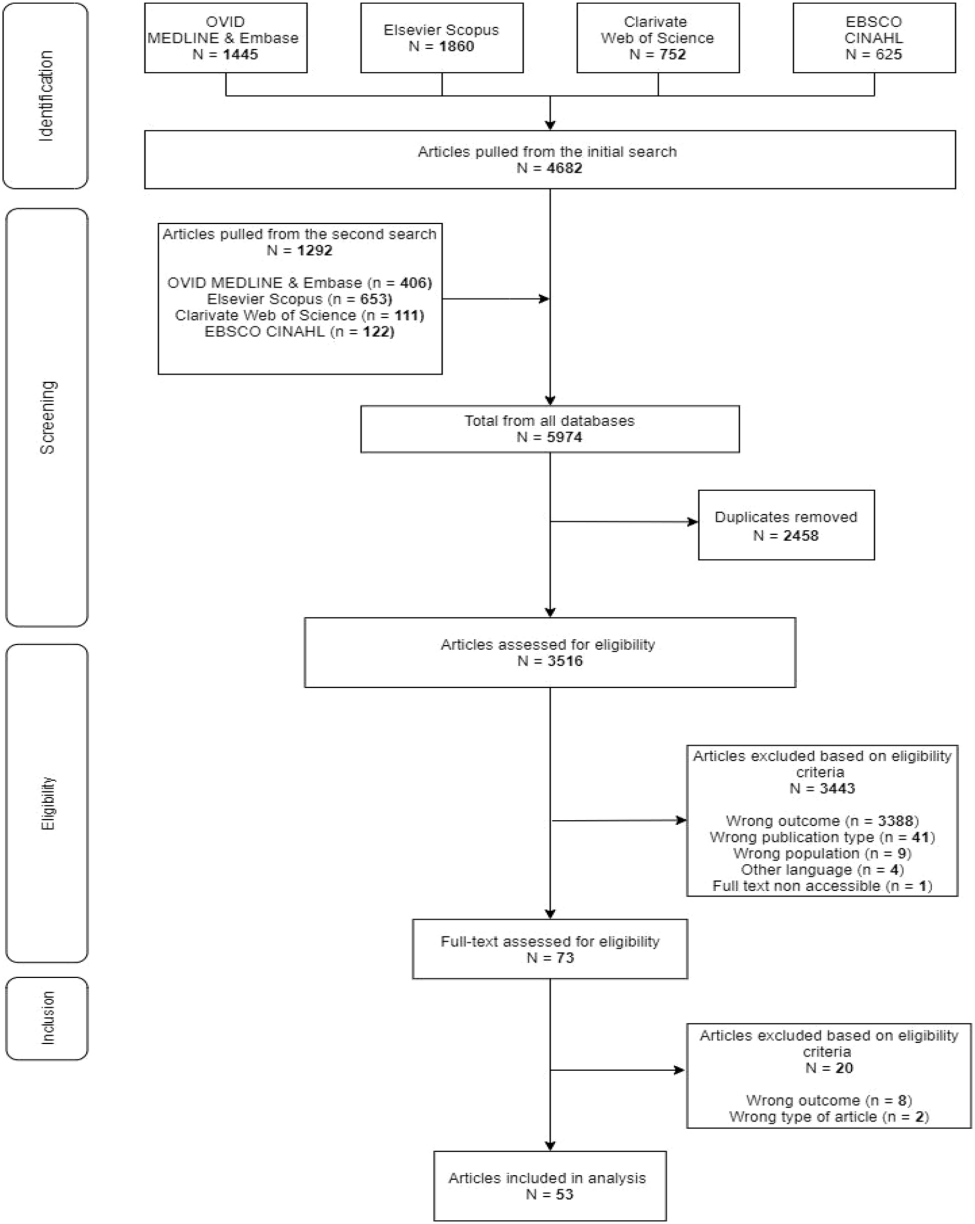
Flowchart of the study selection process

Our search yielded a total of 5974 articles. After removing duplicates, a total of 3516 articles were screened. This process resulted in a total of 73 full text articles to be assessed for eligibility. Following reviews of full-text articles, 53 articles met the inclusion criteria [11, 37–92]. The PRISMA flow diagram has been provided (see Fig. 1).

### Study characteristics

We identified a total of 53 articles based on eligibility criteria for assessing the perceptions of patients living with oncological and non-oncological chronic conditions of therapeutic cannabis, both prescribed and self-purchased. The extraction grid with the identification and analysis of articles has been presented (see Additional file 2). The included studies were published in 45 different journals, from 2003 to 2022 in 14 different countries, with the United States, Canada and Australia being the most represented ones. The chronic conditions studied are presented in (Table 3). Studies collected data through surveys (74%, *n* = 39), interviews (19%, *n* = 10), or focus groups (8%, *n* = 4) (see Additional file 2). Five articles took into consideration sex and age while assessing attitude, methods and dosage of MC consumption. Sixteen articles included a focus on cannabis legislation as a contributing factor in the analysis of medical cannabis consumption patterns and attitudes toward medical cannabis. Sample size ranged from 4 to 2701, with focus groups and interviews having fewer participants than surveys. Conflicts of interests were reported in 13 articles (25%) (see Additional file 2), including: (1) receiving funding, grants, honoraria or personal fees from research pertaining to chronic diseases, organizations or pharmaceutical or MC-related companies; (2) being in a high position (e.g., founder, chief executive officer, board member) or a consultant with those organizations or companies; (3) obtaining free products; (4) having patents pending; (5) being an authorized MC grower and distributor; and (6) reporting data from participants from the same consulting MC company.

**Table 3:** Chronic conditions included in studies

Chronic Conditions	*N*	References
Cancer	17	
Inflammatory bowel disease	4	
Chronic pain	6	
Multiple sclerosis	3	
Chronic conditions at large	3	
Human immunodeficiency virus	2	
Schizophrenia	2	
Spinal cord injury (pain)	2	
Asthma	1	
Cystic fibrosis	1	
Epilepsy	1	
Fibromyalgia	2	
First episode of psychosis	1	
Glaucoma	1	
Migraine and headaches	1	
Post-traumatic stress syndrome	1	
Arthritis	1	
Autosomal recessive spastic ataxia of charlevoix-saguenay	1	
Parkinson disease	1	
Alopecia areata	1	
Primary dysmenorrhea	1	

### Patients’ engagement in research

In terms of patients’ engagement, 12 articles have involved patients in the research process. Five studies (9%) were engaging patients at the first level of involvement (consultation) to identify the main themes to include in the questionnaire (see Additional file 2), four articles involved patients in reviewing the questionnaire once it was created (8%), (see Additional file 2), and four engaged patients for pilot-testing the tool once it was developed (7%) (see Additional File 2). One collaborated with patients by involving them at the beginning (identification of themes) and at the end of the questionnaire creation (feedback on the final version) (see Additional file 2). This can be considered as collaboration, hence the second level of involvement, but not co-creation since patients have not constructed the tool design with researchers and were not involved beyond the tool development stage.

### Perception of MC

Regarding thematic analysis related to how patients’ perception of MC is evaluated in the literature, four main themes were identified: (1) reasons to use, to stop using or not to use MC; (2) beliefs about and knowledge of MC; (3) effects of MC on patients themselves and empowerment; and (4) discussion of MC with relatives and healthcare professionals.

#### Reasons to use, to stop using or not use MC

Of 53 articles, 43 of them studied the motivations behind patients’ decision to use, to stop using or not to use MC (81%) of which 39 inquired about the reasons of use (74%), 13 about why they stopped using MC (25%), and 25 about reasons of non-use (47%) (see Additional file 2). Only five articles asked questions about the three categories (9%) (see Additional file 2).

Reasons cited to use MC include: symptoms’ improvement (47%, *n* = 25), reasons in relation to other medications being taken (e.g., to be used in conjunction with other medications, to reduce the uptake of other medications or because the medication was ineffective; 24%, *n* = 17), quality of life improvement (23%, *n* = 12), reasons in relation to the disease (e.g., coping with the disease, healing or stopping its progression; 21% *n* = 11), information found or discussion (e.g., recommendation from a healthcare provider or a relative, or personal research; 23%, *n* = 12), and ease of access to MC (2%, *n* = 1) (see Additional file 2).

Reasons to stop using MC include: ineffectiveness or loss of interest in using MC (17%, *n* = 9), side effects caused by MC (15%, *n* = 8), concerns about MC (e.g., product legality or security, stigma; 11%, *n* = 6), access difficulties (11%, *n* = 6), and advice from healthcare providers or relatives (8%, *n* = 4) (see Additional file 2).

Reasons not to use MC for treatment of chronic disease include: access difficulties (36%, *n* = 19), concerns about MC (e.g., impact on health, work or social life; 28%, *n* = 15), research missing or lack of information on MC (26%, *n* = 14), advice from healthcare providers or relatives (13%, *n* = 7), and personal choice (13%, *n* = 7) (see Additional file 2).

An overwhelming majority did not consider the legal status of cannabis when assessing the environment to use or not to use MC, and only six articles considered the legality of MC in association with the intention to use MC (see Additional file 2).

#### Perceived effects of MC on patients themselves and empowerment

When assessing perception of MC, 45 articles focused on a biological level and described the results of perceived treatment by studying the effect that MC had on patients living with chronic diseases (85%) (see Additional file 2). Most studies asked patients questions about the effect of MC on their disease’s symptoms (75%, *n* = 40), and some inquired about new perceived side effects on self and symptoms (47%, *n* = 25) and MC effects on quality of life (38%, *n* = 20) (see Additional file 2). Only a few have addressed MC effects compared to other medications (6%, *n* = 3), its effect on the disease progression (9% *n* = 5), the impact on lifestyle habits (e.g., change in their views about MC or in behaviour; 4%, *n* = 2), and the impact on self-image (e.g., affirming self-worth or sense of belonging; 2%, *n* = 1) (see Additional file 2). Fifteen articles studied perceived effects in terms of the empowerment MC had on patients (28%), such as decision-making in their own care or treatment path (e.g., reducing or stopping other medications, preventing other medical interventions, selecting best products for self, or managing MC dose; 21%, *n* = 11), and the will to participate in research activities (e.g., conducting research to educate themselves, or participating in clinical trials; 6%, *n* = 3) (see Additional file 2). Some studies have also noted the positive feelings of empowered patients, such as a comforted feeling for being in control (8%, *n* = 4) (see Additional file 2).

#### Beliefs about and knowledge of MC

Out of the 53 articles included, 41 addressed the patients’ personal beliefs about MC (72%) (see additional file 2). Most inquired about concerns and risk perceptions (53%. *n* = 28), such as the perceived level of security, utility, and efficacy of MC, or concerns about access or product quality (see Additional file 2).

Almost half evaluated the social support or comfort level using MC (42%, *n* = 22) (see Additional file 2). Other elements analyzed included: comparison of MC with other medications (e.g., perception that cannabis is more natural, or safer than other medications, preferences or satisfaction level; 23%, *n* = 12), stereotypes (e.g., belief that MC is addictive, that it is a gateway drug, that its withdrawal can be life-threatening or that its users are prone to violence; 11%, *n* = 6), the impact of media and pharmaceutical companies (e.g., level of influence of the media on their opinion of cannabis, degree of trust in pharmaceutical companies; 6%, *n* = 3), the expected effects on those who plan to use MC (4%, *n* = 2), and the conditions of use of MC (e.g., should only be taken under the guidance of physicians or should be made available to people with qualifying conditions; 4%, *n* = 2) (see Additional file 2).

In addition, 29 articles evaluated the patients’ knowledge of MC (55%). More specifically, questions were related to: information research on MC (e.g., their interest in seeking information, the sources used to find information, the quality of information found, the level of trust in media as a source of information; 38%, *n* = 20), general knowledge of MC (e.g., different cannabis compounds, consumption methods, laws, what it can treat; 23%, *n* = 12), and on MC effects (e.g., benefits and side effects, parts of the brain affected by cannabis; 19%, *n* = 10).

#### Discussion about MC with relatives and healthcare professionals

Twenty-two articles inquired about discussion with the patients’ relatives (42%). Elements of discussion included: concerns about or experienced stigma (19%, *n* = 10), level of comfort discussing MC (17%, *n* = 9), advice received from relatives to use MC (MC recommended by relatives MC; 15%, *n* = 8), level of support received (13%, *n* = 7), and cannabis use by relatives (11%, *n* = 6).

Moreover, 27 articles evaluated patients’ discussions with healthcare professionals (51%). About a third assessed the perceived attitude of the healthcare provider (e.g., reaction, level of support received, perceived openness of the professionals to discuss MC; 30%, *n* = 16), and whether patients have requested information about MC or disclosed their MC use (28%, *n* = 15). The relationship with healthcare professionals, such as the patients’ comfort level discussing taking MC or the level of trust in the healthcare professionals (e.g., decision to prescribe or not, knowledge of the subject) was assessed in 17% of articles (*n* = 9). Other specific elements of discussion (e.g., if they received a prescription, if they asked the professional for advice on how to use the product, who initiated the conversation) were present in 21% of articles (*n* = 8).

## Discussion

The aim of this scoping review was to map out the literature pertaining to how the perception and engagement by people with oncological and non-oncological chronic diseases about MC is evaluated in the literature and how those people are engaged in research related to that topic. Of 53 eligible articles for our analysis, only three have focused on chronic diseases in general, as 50 assessed the perception of patients with a specific chronic condition. As mentioned in the introduction, chronic conditions not only affect a person on a biological level, but also impact their life on a daily basis [3, 35]. Therefore, self-management plays a vital role in patients’ lives and is crucial to longevity and health-related quality of life [7]. As such, our results showed that self-management strategies, including the use of MC, can help patients gain control of the decision-making in their treatment path, and increase their willingness to participate in research activities.

### Thematics identified

#### MC effects and outcomes

When assessing perceptions of MC, the main interest was to capture reasons to use MC. The most common reason to use MC mentioned in the included articles were to improve symptoms, which is consistent with existing literature, where chronic pain is one of the most common conditions to use cannabis for therapeutic reasons [12]. It has been shown that MC can be effective in managing chronic pain [18], and that plant-based cannabis preparations alleviate neuropathic pain. [36] Almost 40% of articles also included quality of life improvements as a reason to use MC, of which some have shown MC to be effective in improving quality of life [19]. MC as having a different status of legality can hinder research on cannabis but also impact the perception of this substance. As research on cannabis is relatively new (the oldest article included is from 2003), there is a need for research with different modalities with respect to diseases, areas with a different legal status of MC, dosing, cannabinoids, and methods of consumption.

Moreover, our results show that the main reason included in articles for patients to stop using MC is ineffectiveness, followed by side effects caused by MC. This topic, however, was covered by only a minority of the included articles. Developing interest in this topic could shed some light on reasons for adherence and observance of treatment. When assessing the perceptions of chronically ill patients of MC, our findings show that this topic is understood in terms of perceived effectiveness. Only 20% of articles have looked at empowerment, decision-making or self-image. It would be pertinent to develop interest in patients' own management of MC, which could help healthcare providers to understand patients’ adherence to and observance of treatment strategies.

Our results show that reasons in relation to other medications taken (either to use MC in conjunction or as a replacement of other medication) and in relation to the disease (e.g., coping with the disease, healing or stopping its progression) are analyzed in the literature as reasons to use MC. It would thus be relevant to further assess why some medications do not work and how joint use of MC and medication affects patients. McCallum and Russo also suggested that clinicians must gain a better understanding of MC dosing and administration methods to maximize therapeutic potential and minimize adverse events. [37]

#### Access to medical cannabis

Only one article cited the ease of access as a reason to use MC, while six articles assessed access difficulties as a reason to stop MC, whereas a lack of access was the main reason analyzed for not using MC. This imbalance shows that it may be pertinent to explore the possibility that access could be a main factor for patients with chronic disease to seek MC usage. Indeed, Kim and Monte [38] showed that cannabis availability and use in Colorado significantly increased after the legalization of marijuana. Moreover, access discrepancy should be further studied, as Walsh and al [39]. found that, in countries where MC is illegal, “authorized” and “unauthorized” users face substantial differences regarding access, and accessing cannabis from illegal markets may increase stigma, legal sanction, and other negative outcomes. Even in countries where MC has been legalized, and public acceptance of cannabis continues to grow, it appears that MC users “remain highly vulnerable to stigma at both interpersonal and institutional levels.” [40] Thus, more efforts should be made to better understand how access affects the decision for people living with chronic conditions to use or not to use MC. Additionally, healthcare providers or informative documents could better explain to patients how to have access to MC, both in countries where MC is legal and illegal.

There is also a need to further explore the relationship between healthcare providers and patients as a barrier to MC access. Indeed, our results show that less than a quarter of articles assessed different elements of relationship (such as the patients’ comfort level in engaging in discussions with healthcare professionals or their level of trust in them), even though studies have shown that some patients “fail to receive appropriate assessment and treatment for a health condition because of being labeled as drug dependent or a pothead” and that stigma about MC can strain the healthcare provider–patient relationship [40, 41]. It is therefore crucial to increase research on the professionals’ level of comfort in discussing MC with patients as well as patients’ comfort and trust level toward their healthcare provider.

#### Medical cannabis education

When studying reasons to stop using MC, we found that the information obtained from personal research or discussion with HCP and relatives accounts for 38%, 51% and 42%, respectively. Surprisingly, those factors are the less studied aspect in the literature. To better understand the decision-making on MC consumption, it is important to further explore how information found on cannabis has an impact in patients’ decision to stop using MC. One reason that may explain this is the low trust level in the media and in pharmaceutical companies. Indeed, our results show that only 6% of articles have assessed their decision to use or not MC according to trust levels and levels of suspicion concerning information shared by the media. This underlines a need for better assessment of these topics and more transfer of knowledge from official sources. Low trust in the media or high suspicion could indicate the need for quality and popularized information available to the public from sources that people can trust.

Another reason that may explain why patients stop using MC can be based on personal biases. Only six articles assess patients’ stereotypes such as preconceived notions about MC regarding addiction, withdrawal, characteristics of MC users (e.g., prone to violence), and risk to lead to stronger drugs. To reduce stigma about MC users, we believe further research must focus on these perceptions to better understand the stereotypes still present in society and to inform the population about them by providing accurate and targeted information. Moreover, as suggested in Bottorf et al. healthcare professionals should receive updated educational training to better guide and advise patients on MC use. [40]

### Patient partnership in research

Our results show that although twelve studies have involved patients into the research process, including one at the second level of the engagement continuum [34], none has co-created the questionnaire with patients. Out of 53 articles, only two assessed patients’ beliefs in a broader manner, including not only the concerns and risks, but also the expected effects. Considering that some patients choose to start using MC based on their personal research, it is important to include every aspect pertaining to the use of MC, to prevent unintentional bias with the information available emphasizing only specific areas of MC consumption and beliefs. Finally, only 4% of the articles (*n *= 2) specifically questioned the patients about the barriers for seeking information from their healthcare professional.

Therefore, including patients in research could help identify crucial elements to include in data collection tools, and help interpret results. This would guarantee that their perspectives contribute essential elements to research findings, aspects that might be neglected otherwise [22].

However, the studies in which patients have been engaged have not relayed how this involvement impacted the quality of methodology. It would be pertinent for future research to analyze how their collaboration with patients impacts research and findings.

### Strengths and limitations

To the best of our knowledge, this article is the first scoping review analyzing which thematics are treated to assess the perception and engagement of patients with chronic conditions with respect to MC. Nevertheless, this review has several limitations. First, the literature research was limited to five databases and papers that were published in English or French only, so relevant studies in other languages might have been missed. We also did not conduct a gray literature search for unpublished data in this area and have excluded preprints that may have held relevant information. We chose to prioritize published and peer-reviewed articles to ensure the quality of the data. We abstain from doing a hand-searching of key journals to find articles that are missing in database and reference list searches, having already identified more than 5000 articles in databases. We also did not update the literature for papers published after 2022. However, some studies had conflicts of interests (*n* = 13), especially regarding the different levels of involvement of some authors in MC companies or in MC-promoting organizations which may have impacted the lens through which their questionnaires were conceptualized, as well as their studies’ outcomes. Second, the fact that we conducted a literature review addressing a broad variety of chronic diseases rather than a specific one, may be considered to be both a strength and a limitation. It portrays a rich overview of the several themes of interest on MC; however, given the fact that most of the articles reviewed in this study focused on one specific disease, it is less evident to establish a general analysis. In addition, the exclusion of studies involving children or adolescents, as well as those related to non-chronic diseases, was a deliberate choice to maintain a focused scope in our current analysis. Therefore, complementary studies to ours might be relevant to enrich the overall understanding of the subject matter regarding these populations. In addition, the contextualization of gender and age as mere background data in the majority of studies poses a challenge to the generalizability of our findings. The selected studies where sex and age have been considered factors in assessing perception represent an underwhelming proportion (9%), while for the majority, it has been contextual data. Moreover, an essential facet that has been absent in the current body of literature is the consideration of ethnicity, which hinders a comprehensive understanding of mechanisms that can play a crucial role in shaping perceptions and attitudes toward MC. Similarly, our findings were limited as few articles considered the legal status of cannabis when assessing the intention to use and attitudes, which could greatly impact perceptions.

## Conclusion

This scoping review has highlighted the fact that very few studies have focused on chronic conditions as a whole, and that none of the tools created to assess patients’ perspectives on MC were co-created with patients living with chronic conditions. With the increasing interest and use of MC, future research should focus on assessing the perceptions of these substances by patients by creating partnerships in research.

Moreover, our results show that the main reason included in articles for patients to stop using MC is ineffectiveness, followed by side effects of MC; such reason was not investigated in most included studies. Therefore, more efforts must be made to understand why cannabis is ineffective in certain patients and how its use affects patients. In addition, as the lack of access was the main reason analyzed for not using MC, more research should be made to better understand how access affects the decision for people living with chronic conditions to use or not to use MC, including the relationship between the healthcare professionals and the patients. Finally, this scoping review demonstrates that few articles have assessed patients’ representation of MC and their trust levels with respect to the media regarding their decision to use or not to use MC. Moreover, we noticed that negative effects of MC when assessing perception were not sufficiently studied and we suggest future research to study those aspects to prevent unintentional bias. We also observed a lack of sufficient data for each chronic condition to conclusively determine whether there exists a distinct perception regarding medical cannabis.

Age, gender, ethnicity of chronically ill patients and the legality of MC were not sufficiently studied. It would be pertinent for future research to assess perception according to those variables to gain a more accurate understanding of MC perceptions’ dynamics in different populations. This underlines a need for a more inclusive research outcome, better quality and accessible information available to the public from reliable sources.

### Supplementary Information

**Additional file 1: **Search strategies.

**Additional file 2: **Extraction grid.

## Data Availability

The datasets generated and/or analysed during the current study are available in the [NAME] repository, [PERSISTENT WEB LINK TO DATASETS].

## Abbreviations

AIDS: Acquired immunodeficiency syndrome
CBD: Cannabidiol
MC: Medical cannabis
PRISMA–SCR: Prisma extension for scoping reviews
THC: Δ^9^-Tetrahydrocannabinol
