# Supplementary material for: Perceptions and engagement of patients with chronic conditions on the use of medical cannabis: a scoping review
Source: Eur J Med Res. 2024 Apr 1;29:211. doi: 10.1186/s40001-024-01803-w (PMC10983766; doi:10.1186/s40001-024-01803-w)
Supplement: Supplementary file 1 — Additional file 1: Search strategies. [file 40001_2024_1803_MOESM1_ESM.docx]

## **Supplemental file 1. Search strategies**

**Ovid-Embase and Ovid-MEDLINE (search date: 29 November 2021)**

| **#** | **Search terms and strategy** |
| --- | --- |
| 1 | (patient* or user* or adult*).ab,ti. |
| 2 | (chronic or pain or disease* or symptom* or illness or condition or disorder*).ab,ti. |
| 3 | (perception* or preference* or attitude* or perspective* or experience* or belief* or view* or opinion*).ab,ti. |
| 4 | (marijuana or cannabis or cannabinoid* or cannabidiol or tetrahydrocannabinol or THC or CBD).ab,ti. |
| 5 | (treatment* or therap* or alternative or management or palliative or complementary or medic*).ab,ti. |
| 6 | (effect* or impact or stigma* or "quality of life" or benefit* or outcome* or harm).ab,ti. |
| 7 | 1 and 2 and 3 and 4 and 5 and 6 |
| 8 | limit 7 to abstracts |
| 9 | limit 8 to ("all adult (19 plus years)" or "young adult (19 to 24 years)" or "adult (19 to 44 years)" or "young adult and adult (19-24 and 19-44)" or "middle age (45 to 64 years)" or "middle aged (45 plus years)" or "all aged (65 and over)" or "aged (80 and over)") [Limit not valid in Embase; records were retained] |
| 10 | limit 9 to human |
| 11 | limit 10 to (adult <18 to 64 years> or aged <65+ years>) [Limit not valid in Ovid MEDLINE(R),Ovid MEDLINE(R) Daily Update,Ovid MEDLINE(R) PubMed not MEDLINE,Ovid MEDLINE(R) In-Process,Ovid MEDLINE(R) Publisher; records were retained] |
| 12 | limit 11 to humans |
| 13 | limit 12 to (english or french) |

**Elsevier-Scopus**

( TITLE-ABS-KEY ( patient* OR user* OR adult* ) AND TITLE-ABS-KEY ( chronic OR pain OR disease* OR symptom* OR illness OR condition OR disorder* ) AND TITLE-ABS-KEY ( perception* OR preference* OR attitude* OR perspective* OR experience* OR belief* OR view* OR opinion* ) AND TITLE-ABS-KEY ( marijuana OR cannabis OR cannabinoid* OR cannabidiol OR tetrahydrocannabinol OR thc OR cbd ) AND TITLE-ABS-KEY ( treatment* OR therap* OR alternative OR management OR palliative OR complementary OR medic* ) AND TITLE-ABS-KEY ( effect* OR impact OR stigma* OR "quality of life" OR benefit* OR outcome* OR harm ) ) AND ( LIMIT-TO ( PUBSTAGE , "final" ) ) AND ( LIMIT-TO ( LANGUAGE , "English" ) OR LIMIT-TO ( LANGUAGE , "French" ) ) AND ( EXCLUDE ( EXACTKEYWORD , "Adolescent" ) OR EXCLUDE ( EXACTKEYWORD , "Child" ) OR EXCLUDE ( EXACTKEYWORD , "Nonhuman" ) OR EXCLUDE ( EXACTKEYWORD , "Animals" ) OR EXCLUDE ( EXACTKEYWORD , "Animal Experiment" ) OR EXCLUDE ( EXACTKEYWORD , "Animal" ) )

**Clarivate - Web of science**

(TI=((patient* OR user* OR adult*) AND (chronic OR pain or disease* OR symptom* OR illness or condition OR disorder*) AND (perception* OR preference* OR attitude* OR perspective* OR experience* OR belief* OR view* OR opinion*) AND (marijuana OR cannabis OR cannabinoid* OR cannabidiol OR tetrahydrocannabinol OR THC OR CBD) AND (treatment* OR therap* OR alternative or management OR palliative or complementary OR medic*) AND (effect* OR impact or stigma* OR “quality of life” OR benefit* OR outcome* OR harm) NOT (animal*) NOT (child* OR adolescent* OR teenager*))) OR (AB=((patient* OR user* OR adult*) AND (chronic OR pain or disease* OR symptom* OR illness or condition OR disorder*) AND (perception* OR preference* OR attitude* OR perspective* OR experience* OR belief* OR view* OR opinion*) AND (marijuana OR cannabis OR cannabinoid* OR cannabidiol OR tetrahydrocannabinol OR THC OR CBD) AND (treatment* OR therap* OR alternative or management OR palliative or complementary OR medic*) AND (effect* OR impact or stigma* OR “quality of life” OR benefit* OR outcome* OR harm) NOT (animal*) NOT (child* OR adolescent* OR teenager*)))

**EBSCO- CINAHL**

(TI=((patient* OR user* OR adult*) AND (chronic OR pain or disease* OR symptom* OR illness or condition OR disorder*) AND (perception* OR preference* OR attitude* OR perspective* OR experience* OR belief* OR view* OR opinion*) AND (marijuana OR cannabis OR cannabinoid* OR cannabidiol OR tetrahydrocannabinol OR THC OR CBD) AND (treatment* OR therap* OR alternative or management OR palliative or complementary OR medic*) AND (effect* OR impact or stigma* OR “quality of life” OR benefit* OR outcome* OR harm) NOT (animal*) NOT (child* OR adolescent* OR teenager*))) OR (AB=((patient* OR user* OR adult*) AND (chronic OR pain or disease* OR symptom* OR illness or condition OR disorder*) AND (perception* OR preference* OR attitude* OR perspective* OR experience* OR belief* OR view* OR opinion*) AND (marijuana OR cannabis OR cannabinoid* OR cannabidiol OR tetrahydrocannabinol OR THC OR CBD) AND (treatment* OR therap* OR alternative or management OR palliative or complementary OR medic*) AND (effect* OR impact or stigma* OR “quality of life” OR benefit* OR outcome* OR harm) NOT (animal*) NOT (child* OR adolescent* OR teenager*)))
